# Association Between BDNF Val66Met Polymorphism and Optic Neuritis Damage in Neuromyelitis Optica Spectrum Disorder

**DOI:** 10.3389/fnins.2019.01236

**Published:** 2019-11-19

**Authors:** Ting Shen, Vivek Gupta, Con Yiannikas, Alexander Klistorner, Stuart L. Graham, Yuyi You

**Affiliations:** ^1^Faculty of Medicine and Health Sciences, Macquarie University, Sydney, NSW, Australia; ^2^Save Sight Institute, The University of Sydney, Sydney, NSW, Australia; ^3^Australia Department of Neurology, Royal North Shore Hospital, Sydney, NSW, Australia

**Keywords:** BDNF, Val66Met, optic nerve damage, NMOSD, optic neuritis

## Abstract

Neuromyelitis optica spectrum disorder (NMOSD) is an autoimmune inflammatory disease of the central nervous system (CNS). The purpose of the study was to examine the association between the brain-derived neurotrophic factor (BDNF) Val66Met genotype and structural and functional optic nerve damage in the eyes of NMOSD patients. A total of 17 NMOSD subjects (34 eyes) were included in the study and were divided into subgroups based on optic neuritis (ON) history and BDNF Val66Met polymorphisms. The mean (range) age was 47.8 (23–78) years, and the mean (SD) disease duration was 7.4 (2–39) years. All participants had undergone optical coherence tomography (OCT) scans for global retinal nerve fiber layer (gRNFL) and ganglion cell-inner plexiform layer (GCIPL) thickness and multifocal visual evoked potential (mfVEP) test for amplitude and latency. BDNF Val66Met polymorphisms were genotyped in all participants. OCT and mfVEP changes were compared between two genotype groups (Met carriers vs. Val homozygotes) by using the generalised estimating equation (GEE) models. The BDNF Val66Met polymorphism was significantly associated with more severe nerve fiber layer damage and axonal loss in ON eyes of NMOSD subjects. Met carriers had more significantly reduced GCIPL (*P* = 0.002) and gRNFL (*P* < 0.001) thickness as well as more delayed mfVEP latency (*P* = 0.008) in ON eyes. No association was found between Val66Met variants and non-ON (NON)-eye of the participants. These findings suggest that the BDNF Val66Met polymorphism may be associated with optic nerve damage caused by acute ON attacks in NMOSD patients.

## Introduction

Neuromyelitis Optica (NMO) is an idiopathic, autoimmune and inflammatory disease of the central nervous system (CNS) that occurs in individuals of all ethnicities. NMO has long been considered as a variant of multiple sclerosis (MS) until the detection of the serum antibodies to the astrocytic water channel aquaporin-4 (AQP4) (Lennon et al., 2004). AQP4 antibodies are detectable in 60–90% of the patients with NMO, but not in the serum of MS patients (Jarius and Wildemann, 2010). This finding together with the foveal thinning reported in NMO patients without optic neuritis (ON) history (Oertel et al., 2017; Yamamura and Nakashima, 2017), supports the concept of NMO to be a predominantly serum AQP4 IgG antibody-mediated astrocytopathy [NMO spectrum disorder (NMOSD)] that is distinct from MS (Wingerchuk et al., 2015). Recently, Müller glial dysfunction has been reported in the eyes of seropositive AQP4-IgG NMOSD patients support a subclinical retinal astrocytopathy in the disease (You et al., 2019). It has been reported that subtype of reactive astrocytes are neurotoxic, which may explain the severe axonal loss observed in NMOSD (Liddelow et al., 2017). MOG-antibody-positive NMOSD has been recently recognized as a possible new inflammatory disorder of the CNS (Jurynczyk et al., 2017), patients with positive MOG-IgG were thus excluded in this study.

A higher frequency of familial NMO cases was observed in a previous study, suggested complex genetic susceptibility in NMOSD (Matiello et al., 2010). Though several genes and genetic variants have been evaluated as contributors to NMOSD, the major genes that confer significant susceptibility are still unknown (Kim et al., 2010; Wang et al., 2011; Wang et al., 2012; Yoshimura et al., 2013; Estrada et al., 2018). Brain-derived neurotrophic factor (BDNF) has unique roles in neuronal growth, differentiation, distribution and survival (Poo, 2001; Gupta et al., 2014). The genetic variation that is the focus of this study has a single nucleotide substitution from G to A (Val66Met, NCBI database dbSNP rs6265) in the pro-region of the BDNF gene. The BDNF Val66Met polymorphism has been associated with various neurodegenerative and psychiatric diseases (Notaras et al., 2015; Shen et al., 2018b). In a recent study, carriage of Met alleles was associated with lower rates of optic nerve damage and was reported to be reducing the long-term open-angle glaucoma (OAG) progression in female patients (Shen et al., 2019). However, it remains to be elucidated whether Val66Met is associated with neuroaxonal damage in NMOSD. Optical coherence tomography (OCT) has been used to examine rates of axonal damage through retinal nerve fibre layer (RNFL) and macular thickness in NMO and MS which could help distinguish the patterns of optic nerve damage in the two diseases (Naismith et al., 2009; Ratchford et al., 2009). Multifocal visual evoked potentials (mfVEPs) provide functional assessments of axonal loss (amplitude) and demyelination (latency) in the visual pathway (Klistorner et al., 2010). NMOSD patients were suggested to have a more severe axonal loss in ON eyes by more significantly reduced mfVEP amplitude compared to MS (Shen et al., 2018a). The aim of the present study was to clarify whether the carriage of Met allele is associated with axonal loss and demyelination in ON and non-ON (NON) eyes of NMOSD participants by using both structural (OCT) and functional (mfVEP) analysis.

## Methods

Neuromyelitis optica spectrum disorder (*N* = 17) patients were recruited from four tertiary neuro-ophthalmology or neurology clinics in Sydney. The NMOSD diagnosis was made per the 2015 diagnostic criteria (Wingerchuk et al., 2015). Exclusion criteria are: patients tested as MOG-positive, retinal, optic nerve, or other neurologic diseases affecting the visual system, including any other types of optic neuropathy, optic atrophy with no known cause. Patients tested within 12 months of the acute ON were also excluded from the study to eliminate the decline of amplitude and prolongation of latency in mfVEP in a post-acute stage of ON (Klistorner et al., 2010). Two participants with visual acuities worse than hand movement were not able to complete mfVEP testing and were excluded from mfVEP analysis. Details on the recruitment, composition and selective attrition are described in detail elsewhere, blood samples from all but two participants with MOG antibodies in the previous study were obtained (Klistorner et al., 2010). The ethnic background of the participants is mainly Caucasian, except for one Asian subject. All participants underwent OCT scans for RNFL and ganglion cell-inner plexiform layer (GCIPL) thickness, multifocal VEP testing for amplitude and latency, and MRI scans for whole-brain (WB) lesion volume (LV) as previously reported.

The study was conducted in accordance with the Declaration of Helsinki and approved by the Human Research Ethics Committee of the University of Sydney (Sydney, NSW, Australia). Written informed consent was obtained from all study participants.

### Genotyping

The genomic DNA was isolated from peripheral blood with a commercially available DNA extraction kit (Qiagen, Hilden, Germany). Quantification of isolated DNA was carried out with a spectrophotometer (Thermo Scientific, Rockford, IL, United States). The G → A nucleotide substitution, identifying the of BDNF Val66Met polymorphism, was assayed by polymerase chain reaction (PCR, Eppendorf, Hamburg, Germany). The primers used in this study were as follows: forward 5′ ACTCTGGAGAGCGTGAATGG 3′ and reverse 5′ TCCAGGGTGATGCTCAGTAGT 3′. The amplification conditions for PCR were initiated at 95°C for 5 min, followed by 30 cycles comprising of denaturation at 94°C for 1 min, annealing at 55°C for 30 s and extension at 72°C for 1 min, with a final extension step of 5 min at 72°C. The carriage of BDNF Val66Met polymorphism was determined by direct sequencing (both directions, Australian Genome Research Facility, Sydney, NSW, Australia) of genomic DNA. A detailed description of the genotyping procedure is provided elsewhere (Shen et al., 2018b).

### Statistical Analysis

Statistical analysis was performed using SPSS software version 22 (SPSS, Inc., Chicago, IL, United States). Data from both ON eyes and NON eyes were included for the comparison between two genotype groups using the generalised estimating equation model (GEE). The gender was included as a cofactor, and the age, disease duration and number of ON episodes of all patients were included as covariates in the GEE analysis. The correlation between OCT and mfVEP parameters were also examined using the GEE method with adjustment of intrasubject factors. A *P*-value of less than 0.05 was considered statistically significant, and a *P*-value between 0.05 and 0.1 was considered borderline significant.

## Results

Demographic and clinical features of the participants included in the study are presented in Table 1. Twenty eyes of 17 patients with NMOSD had ON history and was included in the following GEE analysis as ON eyes. Fourteen eyes of the patients had no previous ON attacks and were included as NON-eyes in the statistical analysis. The mean age of the patients was 47.8 years (range, 23–78). No significant difference was found in sex ratio, AQP4 status, age, disease duration, BCVA or T2 WB LV between two genotype groups (Table 1).

**TABLE 1:** Demographic and clinical characteristics of participants.

	**Total (*N* = 17)**	**Val/Val (*N* = 9)**	**Met carriers (*N* = 8)**	***P*-values**
Eyes, *N* (%)	34	18 (52.9)	16 (47.1)	n.a.
ON eyes, *N* (%)^a^	20 (58.8)	10 (55.6)	10 (62.5)	0.68
NON-eyes, *N* (%)^a^	14 (41.2)	8 (44.4)	6 (37.5)	
ON history, *N* (%)	13 (76.5)	6 (66.7)	7 (87.5)	0.31
Unilateral ON history, *N* (%)^a^	6 (35.3)	2 (22.2)	4 (50.0)	0.39
Bilateral ON history, *N* (%)^a^	7 (41.2)	4 (44.4)	3 (37.5)	
Female, *N* (%)^a^	11 (64.7)	6 (66.7)	5 (62.5)	0.86
AQP4-IgG seropositive, *N* (%)^a^	13 (76.5)	6 (66.7)	7 (87.5)	0.31
Age, years, mean (range)^b^	47.8 (23–78)	42.8 (23–64)	53.4 (38–78)	0.14
Disease duration, years, mean (SD)^b^	7.4 (10.1)	10.9 (13.2)	3.5 (1.9)	0.1
BCVA, LogMAR, mean (SD)^b^	0.4 (0.8)	0.3 (0.8)	0.4 (0.9)	0.63
T2 WB LV, cm^3^, mean (SD)^c^	860.2 (1858.0)	926.0 (1737.7)	786.3 (2104.2)	0.91

*AQP4-IgG = astrocyte water channel aquaporin-4 immunoglobulin G; BCVA = best-corrected visual acuity; WB = whole brain; LV = lesion volume. Statistics: ^*a*^chi-square test; ^*b*^generalized estimating equation; ^*c*^Mann–Whitney *U* test. Neuromyelitis optica spectrum disorder treatment: immunosuppressants (*n* = 13, including azathioprine, mycophenolate, rituximab, and prednisolone), plasma exchange (*n* = 3), and not receiving treatment (*n* = 3). No parameter showed significant differences between two genotype groups.*

The genotype frequencies of Val66Met among the NMOSD cohort were 9 (52.9%) for genotype GG (Val/Val), 7 (41.2%) for GA (Val/Met), and 1 (5.9%) for AA (Met/Met). The allele frequency of the A allele (Met) was 26.5% in the study subjects.

The OCT and mfVEP measures were compared between two genotype groups and are summarized in Table 2. In ON eyes, there were significant differences between the BDNF Val66Met polymorphism genotypes in averaged GCIPL thickness (*P* = 0.002) and global retinal nerve fiber layer (gRNFL) thickness (*P* < 0.001). In parallel with the OCT changes, carriage of Met allele was also associated with significantly more prolonged latency (*P* = 0.008) in ON eyes. The reduction of mfVEP amplitude showed the borderline difference between Met and Val genotypes (*P* = 0.07) in ON eyes. In NON-eyes, no significant difference was observed in any of the parameters mentioned above (Table 2). As there are possibilities for the AQP4 seronegative patients to be affected by another disease (Jarius et al., 2012), a sub-analysis was performed to validate the differences in ON eyes and excluded the potential influence of AQP4 serology. In AQP4 seropositive patients (16 eyes), significant differences were observed between genotype groups in GCIPL, gRNFL and mfVEP latency (*P* < 0.001, *P* = 0.04, and *P* < 0.001, respectively). There was also a borderline significant difference between Val/Val and Met carriers in mfVEP latency (*P* = 0.1).

**TABLE 2:** Association between BDNF Val66Met polymorphism and optic nerve damage in NMOSD.

**Parameters**			**Val/Val**	**Met carriers**	**Difference**		***P*-values**
						
					**Value**	**95% CI**	
ON eyes	OCT	GCIPL	62.19	59.43	2.76	0.97 to 4.55	0.002^∗^
		gRNFL	82.23	58.84	23.39	20.54 to 26.25	<0.001^∗^
	mfVEP	Amp	137.64	68.54	69.1	−4.67 to 142.86	0.07
		Lat	152.13	163.39	−11.26	−19.61 to−2.91	0.008^∗^
NON-eyes	OCT	GCIPL	78.81	75.44	3.37	−9.13 to 15.88	0.6
		gRNFL	98.98	95.2	3.78	−22.72 to 30.28	0.78
	mfVEP	Amp	171.45	184.71	−13.26	−46.57 to 20.06	0.44
		Lat	148.91	149.87	−0.96	−7.32 to 5.40	0.77

*ON = optic neuritis; OCT = optic coherence tomography; GCIPL = ganglion cell-inner plexiform layer; gRNFL = global retinal nerve fiber layer; mfVEP = multifocal visual evoked potential; NON = non-optic neuritis, n.s. = not significant. GEE models were applied assuming additive effect for the minor allele variant (Met carriers), adjusted for sex, age, disease duration, and ON episodes. Statistical significance was defined as *P* < 0.05 an asterisk (^∗^).*

**FIGURE 1:**
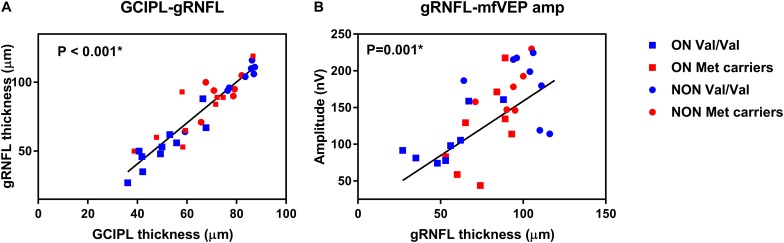
Data visualization showing correlation analysis of **(A)** ganglion cell-inner plexiform layer (GCIPL) and global retinal nerve fiber layer (gRNFL) and **(B)** gRNFL and multifocal visual evoked potential (mfVEP) in neuromyelitis optica spectrum disorders (NMOSD).

The correlation between GCIPL and gRNFL (*P* < 0.001) was showed in Figure 1A as data visualization. In addition, correlation analysis demonstrated strong positive correlations in NMOSD participants with increased GCIPL (*P* < 0.001) and gRNFL (*P* = 0.001) thickness associated with higher mfVEP amplitude (Figure 1B). There was a significant correlation between GCIPL and mfVEP latency (*P* = 0.03). A borderline significant correlation was also observed between gRNFL and mfVEP latency (*P* = 0.06).

## Discussion

This study suggests that BDNF Val66Met polymorphism may be associated with more severe ON attacks in NMOSD patients. In cross-sectional analysis, the Met carriers were found to have significantly more reduced GCIPL and gRNFL thickness, more diminished mfVEP amplitude (borderline significance) and significantly more delayed mfVEP latency than Val homozygotes in ON eyes. The genotypic effect of Val66Met on NMOSD remains positive even with adjustment for a range of cofactors and covariates, including sex, age, disease duration, and ON episodes. In contrast, no association was found between BDNF polymorphisms and changes in NON-eyes of patients. Correlation analysis suggested positive associations between structural and functional measurements in the NMOSD cohort. To our knowledge, this is the first study to suggest that the presence of Met alleles may convey higher rates of axonal damage in ON in NMOSD. The frequency of the BDNF Val66Met polymorphism varies among different ethnicity. In our cohort, the Met allele frequency is 26.5%, which is close to the frequencies previous reported in the United States (28.0%) (Parsian et al., 2004) and Italian cohorts (29.7%) (Ventriglia et al., 2002). OCT is an objective, simple and non-invasive method that allows *in vivo* measurement of the thickness of retinal nerve fibers. It has been reported that the actual RNFL thickness measured from histologic sections would be 4–12% larger than the corresponding OCT measurements of nerve fibers (Toth et al., 1997). Previous studies demonstrated that ON in NMO is associated with thinner RNFL, GCIPL, and mfVEP amplitude compared to MS (Naismith et al., 2009; Shen et al., 2018a). The mfVEP amplitude and latency were suggested to be highly correlated with RNFL thickness (Parisi et al., 1999; Trip et al., 2005; Klistorner et al., 2008).

The age distribution was suggested to influence the AQP4 autoimmunity where females aged 65 years above were more likely to be seropositive than other age groups, and the detection rate of AQP4-IgG rise exponentially in females after 50 years old (Quek et al., 2012). A recent study has also established that the gray matter atrophy and RNFL thinning were correlated with longer disease duration in NMOSD (von Glehn et al., 2014). There were no significant differences between two genotype groups in gender, age or disease duration in this study, and though all these parameters have been included and adjusted for as either cofactors or covariate, the results remained significant in ON eye analysis. It has been reported that BDNF is transported anterogradely and retrogradely, and BDNF secretion has a selective effect on the survival and maintenance of lesioned optic nerve axons (Weibel et al., 1995; Fawcett et al., 1998). As the carriage of BDNF Val66Met polymorphism has been associated with reduced activity-dependent secretion of BDNF (Egan et al., 2003), the more severe axonal loss in ON eyes may be explained by less availability of BDNF within the retina and along the higher visual pathway.

The results of the present study demonstrated a potential negative effect BDNF Val66Met polymorphism has on ON damage in NMOSD, which contrasts with the previous glaucoma study where the Met alleles exhibited protective effects on OAG progression in a gender-specific way (Shen et al., 2019). The opposite effect may suggest different mechanisms in the axonal loss in glaucoma and NMOSD. The precursor form of BDNF (pro-BDNF) was suggested to increase cell apoptosis, while in contrast, mature BDNF (mBDNF) enhances cell survival (Lu et al., 2005). In neurons, pro-BDNF is generally converted to mBDNF by proteases such as tissue plasminogen activator (tPA), and cerebral tPA expression was reported to decrease with age in an animal model (Cacquevel et al., 2007). Thus, higher levels of BDNF secretion in Val/Val may not increase neuronal function unless there are enough cleavage molecules to convert BDNF from the precursor form to mature form. The mean age was approximately 70 for the participants in the glaucoma study, and only 47 in our NMOSD cohort, the significant age difference between the cohorts may contribute to differentiated tPA levels and opposite effects of Val66Met on RGC survival. In addition, it is well established that in glaucoma, elevated intraocular pressure is the main cause of RGC apoptosis and optic nerve head degeneration via several mechanisms (Kuehn et al., 2005), while in NMOSD, inflammation of the optic nerve and spinal cord were usually dominating the disease and causing axonal damage. It has been reported that BDNF has protective effects against ischemic insult by modulating local inflammation in the brain in an animal model (Jiang et al., 2011). The Met allele may interact differently with acute optic nerve damage in inflammation and progressive glaucomatous optic neuropathy; however, future studies are warranted to investigate further the cellular mechanisms of the contrast effects BDNF Val66Met have in two diseases.

There are several potential limitations to the current study. First, the sample size of NMOSD is relatively small due to the rarity of the disease in the Caucasian population. However, the genotype distribution was favorable with both eyes included in the analysis, which significantly increased the sample size and met the minimum requirement for desired statistical power. The *P*-values were especially small for both gRNFL and GCIPL thickness (Table 1), which were likely representing true differences between the two genotype groups. Second, the investigation was in the absence of a healthy control group. This study is also limited by the cross-sectional study design, future longitudinal study with larger sample size is necessary to validate the reproducibility of the results, especially a cohort with only AQP4 seropositive subjects to exclude the possibility of seronegative patients influenced by another disease. In addition, future research could also focus on the BDNF Val66Met polymorphism’s effect on ON in other neuroinflammatory diseases such as MS.

## Conclusion

Our study demonstrated significant associations between carriage of BDNF Met allele and more severe axonal damage in ON eyes in NMOSD patients. The difference was not found in NON-eyes of participants indicating the effect of BDNF Val66Met on RGC axonal loss in NMOSD is specifically related to acute inflammatory ON attacks.

## Data Availability Statement

The raw data supporting the conclusion of this manuscript will be made available from the corresponding author, without undue reservation, to any qualified researcher.

## Ethics Statement

The studies involving human participants were reviewed and approved by Human Research Ethics Committee of the University of Sydney (Sydney, NSW, Australia). The patients/participants provided their written informed consent to participate in this study.

## Author Contributions

TS: acquisition, analysis and interpretation of data, and drafting and revising the work. VG: revising the work and acquisition of data for the work. CY: acquisition of data and design of the work. AK and YY: design of the work, revising the work, and provided approval for publication of the content. SG: revising the work and provided approval for publication of the content.

## Conflict of Interest

The authors declare that the research was conducted in the absence of any commercial or financial relationships that could be construed as a potential conflict of interest.
